# GIGANTEA supresses wilt disease resistance by down-regulating the jasmonate signaling in *Arabidopsis thaliana*

**DOI:** 10.3389/fpls.2023.1091644

**Published:** 2023-03-09

**Authors:** Alena Patnaik, Aman Kumar, Anshuman Behera, Gayatri Mishra, Subrat Kumar Dehery, Madhusmita Panigrahy, Anath Bandhu Das, Kishore C. S. Panigrahi

**Affiliations:** ^1^ School of Biological Sciences, National Institute of Science Education and Research (NISER) Bhubaneswar, Jatni, Odisha, India; ^2^ Homi Bhabha National Institute (HBNI), Training School Complex, Anushakti Nagar, Mumbai, India; ^3^ Department of Botany, Utkal University, Vani Vihar, Bhubaneswar, Odisha, India

**Keywords:** GIGANTEA, *Arabidopsis thaliana*, *Fusarium oxysporum*, plant-pathogen interaction, salicylic acid, jasmonic acid, circadian clock, flowering

## Abstract

GIGANTEA (GI) is a plant-specific nuclear protein that plays a pleiotropic role in the growth and development of plants. GI’s involvement in circadian clock function, flowering time regulation, and various types of abiotic stress tolerance has been well documented in recent years. Here, the role of GI in response to *Fusarium oxysporum* (*F. oxysporum*) infection is investigated at the molecular level comparing Col-0 WT with the *gi-100* mutant in *Arabidopsis thaliana*. Disease progression, photosynthetic parameters, and comparative anatomy confirmed that the spread and damage caused by pathogen infection were less severe in *gi-100* than in Col-0 WT plants. *F. oxysporum* infection induces a remarkable accumulation of GI protein. Our report showed that it is not involved in flowering time regulation during *F. oxysporum* infection. Estimation of defense hormone after infection showed that jasmonic acid (JA) level is higher and salicylic acid (SA) level is lower in *gi-100* compared to Col-0 WT. Here, we show that the relative transcript expression of *CORONATINE INSENSITIVE1* (*COI1*) and *PLANT DEFENSIN1.2* (*PDF1.2*) as a marker of the JA pathway is significantly higher while *ISOCHORISMATE SYNTHASE1* (*ICS1*) and *NON-EXPRESSOR OF PATHOGENESIS-RELATED GENES1* (*NPR1*), the markers of the SA pathway, are downregulated in the *gi-100* mutants compared to Col-0 plants. The present study convincingly suggests that the GI module promotes susceptibility to *F. oxysporum* infection by inducing the SA pathway and inhibiting JA signaling in *A. thaliana*.

## Introduction

The transition from a vegetative to a reproductive phase in plants is influenced by several factors including light, temperature, age, and hormones. Photoperiodic flowering occurs when this transition is influenced by light and fine-tuned by diurnal and circadian regulatory mechanisms (de Montaigu et al., 2010; Sanchez and Kay, 2016). GIGANTEA (GI) is one of the crucial circadian clock output components in plants (Fowler et al., 1999; Kim et al., 2013). GI gene was first discovered in *Arabidopsis* because mutations in it delayed flowering response to inductive photoperiods with little variation in short days (SDs) (Rédei, 1962; Fowler et al., 1999). In *Arabidopsis thaliana*, a single copy of GI contains 14 exons that code for a 127-kDa protein with 1,173 amino acids (Park et al., 1999). GI is reported to be a nuclear protein with a nuclear localization sequence between residues 543 and 783 in the core 241-amino-acid region (Huq et al., 2000). GI structural homology reveals that it lacks any conserved protein domains (David et al., 2006; Mishra and Panigrahi, 2015). GI is diurnally regulated, and its transcript and protein levels are strictly controlled by the circadian clock (Patnaik et al., 2022). Any change in the components of the circadian clock has been demonstrated to alter the transcription of GI. Furthermore, despite overexpressing the GI protein with a constitutive promoter, its abundance follows a cyclic pattern of aggregation under long days (LDs) and SDs, indicating an additional layer of regulation on the protein expression at the post-transcriptional level (David et al., 2006; Yu et al., 2008). The ubiquitin E3 ligase activity of CONSTITUTIVE PHOTOMORPHOGENIC 1 (COP1) controls the post-transcriptional regulation of the GI protein, which leads to its degradation by the 26S proteasome machinery.

In vascular plants, GI is evolutionarily conserved and plays an important role in a variety of physiological responses. The role of GI in photoperiodic flowering has been thoroughly studied, where it is required to increase the transcription of *CONSTANS* (*CO*) and *FLOWERING LOCUS T* (*FT*) (Fornara et al., 2009). The degradation of repressors from the DOF family of transcriptional regulators, known as CYCLING DOF FACTORS (CDFs), is necessary for the stimulation of CO and FT expression under LD conditions. In the afternoon, GI forms a complex with the blue light-absorbing FLAVIN-BINDING KELCH REPEAT F-BOX 1 (FKF1), an F-box protein, which degrades the DOF transcriptional repressors, allowing *CO* transcription leading to flowering under LD conditions (Imaizumi et al., 2005; Sawa et al., 2007; Kim et al., 2013). Other blue light circadian photoreceptors like LOV KELCH protein 2 (LKP2) and ZEITLUPE (ZTL) interact and stabilize GI (Kim et al., 2007). This regulates the appropriate development and resilience of the circadian clock in plants (Somers et al., 2000; Kim et al., 2007; Fornara et al., 2009; Baudry et al., 2010). In recent times, GI has been shown to act as a co-chaperone in *A. thaliana*’s circadian clock, facilitating the maturation of ZTL (Cha et al., 2017).

Different abiotic stresses, such as cold, drought, salt, and oxidative stress, are regulated by GI in the plants. The level of GI transcript is increased during cold stress, inducing cold acclimation in *A. thaliana via* a C-repeat binding protein (CBF)-independent route (Fowler and Thomashow, 2002). In contrast, another finding states that cold responsive gene (COR) mRNA expression is higher in *gi* mutants than in Col–0 WT, and this is likely to be responsible for promoting freezing tolerance in *gi* mutant plants, implying an additional overlay in regulating transcription of the cold response pathway in *gi* mutant plants (Fornara et al., 2015). GI is shown to play a crucial role in drought tolerance too. Drought resistance is improved by GI *via* the abscisic acid (ABA)-dependent drought escape mechanism (Riboni et al., 2013) besides its inhibitory role in salinity stress tolerance. It inhibits Salt Overly Sensitive (SOS1) and Na^+^/H^+^ anti-porter channels by binding directly to SOS2. Furthermore, while salt stress has no effect on GI transcription, it reduces the protein stability (Kim et al., 2013). When it comes to oxidative stress tolerance, the GI functions as an inhibitor (Riboni et al., 2013). The absence of GI promotes the constitutive production of *SUPEROXIDE DISMUTASE* (*SOD*) and *ASCORBATE PEROXIDASE* (*APX*) genes, and therefore, *gi* mutants are resistant to oxidative stress caused by H_2_O_2_ (Riboni et al., 2013). Although *gi* mutants have been reported to provide resistance to *Fusarium oxysporum* (*F. oxysporum*) infection compared to wild-type plants in *A. thaliana* , the molecular mechanism is still unknown.

*F. oxysporum* is a soil-borne hemi-biotrophic fungal pathogen, which is the causative pathogen for vascular wilt disease in plants (Michielse and Rep, 2009; Lyons et al., 2015a). It begins its disease cycle as a biotroph, infecting the root system, which gradually spreads up to the vasculature, where it secretes phototoxic chemicals, causing wilting. This necrotrophic pathogen eventually causes necrosis, plant senescence, and death (Czymmek et al., 2007; Cole et al., 2014; Lyons et al., 2015a). To combat pathogen invasion, plants use a variety of defense mechanisms. The host response to *Fusarium* infection has been found to be regulated by ABA, ethylene, and auxin that improve vulnerability to this disease, while gibberellic acid (GA) was thought to influence jasmonic acid (JA)/salicylic acid (SA) signaling (Anderson et al., 2004; Kidd et al., 2011; Pantelides et al., 2013). In *A. thaliana*, the jasmonate (JA) signaling pathway contributes both positively and negatively to resistance against *F. oxysporum*. JA receptor mutants like *coi1* have evoked resistance to *F. oxysporum* (Thatcher et al., 2009), but bioactive jasmonates produced during infection have been found to cause senescence of the host (Cole et al., 2014). SA improves resistance to *F. oxysporum* during the biotrophic phase of infection (Edgar et al., 2006; Cole et al., 2014). SA-deficient *sid2* mutant is more susceptible to *Fusarium* wilt than the WT (Diener and Ausubel, 2005). Late-flowering mutants, such as *gi-1*, *mediator 8* (*med8*), *phytochrome and flowering time1* (*pft1*), *auxin response factor 2* (*arf2*), and *myc2*, have also been found to promote resistance to *F. oxysporum* in plants (Kidd et al., 2009; Gangappa and Chattopadhyay, 2010; Lyons et al., 2015a; Lyons et al., 2015b), implying a role of flowering genes in modulating the defense pathways in plants. Despite plenty of studies that describe possible functions of GI in abiotic stress response, little is known about its involvement in biotic stress tolerance (Lyons et al., 2015a; Kundu and Sahu, 2021; Singh, 2022).

Biotic stress is also reported to be important in modulating the flowering transition (Kazan and Lyons, 2016), pointing to a possible link between GI and biotic stress tolerance. Thus, exploration on the role of GI on disease defense mechanism of plant is very important in global climate changing scenario. We demonstrated the role of GI in *F. oxysporum* induced biotic stress tolerance of *A. thaliana* through transcript expression analysis of JA and SA pathway genes in *gi-100* mutants besides confocal microscopy of root cortical cell morphology, stomatal anomalies during fungal progression, disease severity, PS II activity changes in relation to Chl *a* fluorescence, and thermal imaging of internal leaf temperature behaviors in the presence and absence of GI.

## Materials and methods

### Plant growth conditions

**Figure 1:**
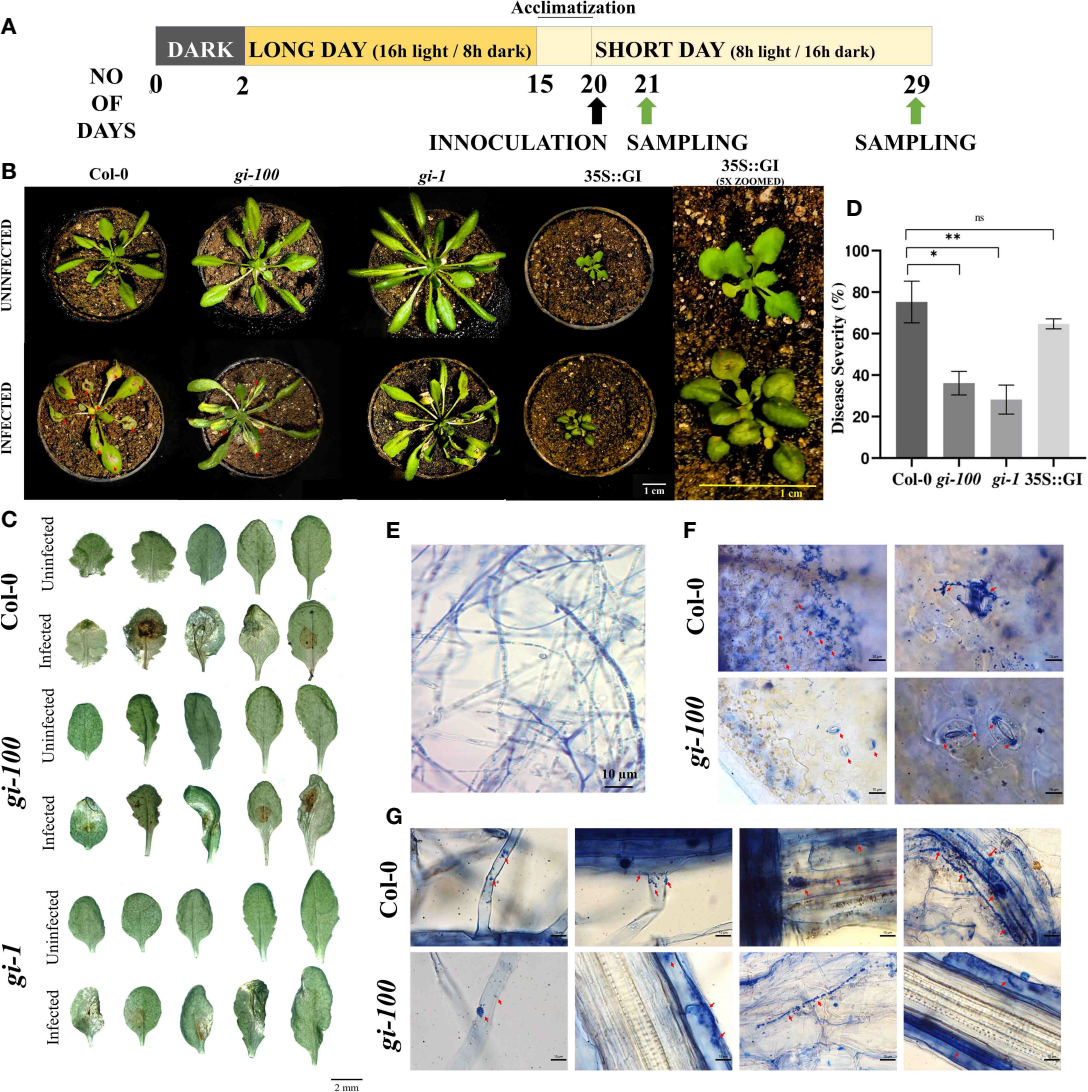
Effect of *F. oxysporum* infection on GIGANTEA (GI) in *Arabidopsis thaliana*. **(A)** Schematic representation of light treatment, plant growth conditions, and sample harvest. **(B)** Plant in pots infected with wilt disease caused by *F. oxysporum*. Columbia-0 (Col-0), *gigantea* mutant (*gi-100, gi-1*), and overexpressed GI (35S::GI) plants after 9 and 5 days post inoculation (DPI), respectively. **(C)** Leaf morphology of Col-0, *gi-100*, and *gi-1* mutant plants after 9 DPI infected with *F. oxysporum*. The leaves of infected and uninfected plants were excised 9 DPI and were photographed using a Carl Zeiss stereo microscope. **(D)** Disease progression after *F. oxysporum* inoculation in Col-0 and *gi-100* was interpreted as in disease severity percentage (area in square meters of necrosis). The disease severity was analyzed using ImageJ software as described in *Materials and methods*. **(E)** Viability of fungal hyphae of *F. oxysporum* was reclaimed by staining with trypan blue before infection. **(F)** Fungal invasion in Col-0 and *gi-100* stained with trypan blue in leaf mesophyll cells in the upper row and lower row, respectively. Magnification is indicated on the top right of each figure. **(G)** Progression of fugal hyphae from lateral hair cells to tap root cells (left to right) in Col-0: upper row and *gi-100*: lower row visualized at 100×. Data presented as mean ± SE. Student’s paired *t*-test was used to evaluate significant differences and compared between Col-0 and *gi-100* plants. *p < 0.05, **p < 0.01, ns, Non-significant.

The seeds of *A. thaliana* Columbia 0 (Col-0) ecotype, mutant, and overexpressor lines of GI, i.e., *gi-100*, *gi-1*, GI::GI-TAP, and 35S::GI (Berns et al., 2014), respectively, were vernalized at 4°C for 48 h in pots containing soil followed by growth under white light in controlled plant growth chambers (Model No-AR36, Percival, USA) until the end of the experiment. As all the genotypes used in this study vary substantially in their growth, morphology, and flowering time (Mishra and Panigrahi, 2015), an initial growth period of 15 days under LD conditions (i.e., 16 h light and 8 h dark, 22°C) was adapted (**Figure 1A**) to obtain a synchronized growth morphology among them with comparable number of rosette leaves (**Figure 1B**, upper panel) before fungal inoculation. On day 16, plants were shifted to SD conditions (8 h light and 16 h dark, 22°C) and all the sampling as well as experiments were done under SD conditions to focus the study mainly in the vegetative stage of the plant (**Figure 1A**). An entrainment period of 5 days was imposed immediately after shifting the plants to SD before the fungal inoculation on 20 days. At the time of infection, all the three genotypes (Col-0, *gi-100*, and *gi-1*) had nearly 14 ± 2 rosette leaves (**Figure 1B**).

### *F. oxysporum* infection and pathogenesis

The pure culture of *F. oxysporum* was taken by aseptically punching out 5 mm of *F. oxysporum* fungal colony from stock. It was allowed to grow for 15 days in a 250-ml conical flask containing 100 ml of potato dextrose broth (PDB) along with the inoculum, in a shaker maintained at 25°C and 60 rpm (Patnaik et al., 2022). After the incubation period, the culture and the fungal mycelia were separated using a sterile nylon cloth, and the density of the conidial suspension was counted using a hemocytometer until it reached a concentration of ~ 6.5 ×10^6^ spores/ml. Few drops of this suspension culture were taken and stained with trypan blue solution to ensure the viability and to visualize the structure of fungal hyphae (**Figure 1E**).

Three-week-old plants were infected with the *F. oxysporum* culture either at the roots by dropping ~200 µl of the conidial suspension in pots or ~10 µl of inoculum on the adaxial surface of the leaves at dawn. Plants with a similar amount of PDB medium without fungal culture was used as the control. To better ensure the pathogenicity, the infection was induced both in the root and leaves of the plant. A similar method of leaf infection caused by *F. oxysporum* has already been reported in Wang et al. (2021). Incidences of the spread of the disease as necrotic lesions were first observed on the fifth day of infection (data not shown). Leaves from infected and uninfected plants were sampled 24 hours post infection (HPI) and 9 days post infection (DPI) at ZT-0, as many defense-related genes were highly induced at dawn (de Leone et al., 2020). The experiments were repeated three times with similar results.

The leaves of the infected and uninfected plants after 9 DPI were studied using a Carl Zeiss stereo microscope (Model number: Stereo Discovery. V20) equipped with 5.8× magnification and an Axiocam 305 camera to obtain the total area of infection. Disease severity percentage was calculated using ImageJ (version: ImageJ 1.53a). Ten different morphologically similar leaves were chosen from six plants of each category to analyze the disease severity. The experiment was repeated three individual times.

### RNA extraction and transcript level validation

Plant tissues (roots and leaves) were snap frozen with liquid nitrogen and immediately stored at −80°C until further use. Total RNA was extracted from ~500 mg of plant samples using the RNeasy Plant Mini Kit (supplied by Qiagen, Cat #74136). One microgram of total RNA was used to obtain cDNA using Reverse Transcription Super-mix (supplied by Bio-Rad, Cat #1708840). For relative transcript levels, quantitative RT-PCR (qRT-PCR) was carried out using the Bio-Rad laboratories’ CFX384 Touch™ Real-time detection system, following the manufacturer’s instructions and iTaq™ universal SYBR green super mix as done previously (Kumar, 2021). Primer Quest tool (Integrated DNA Technologies, Inc., USA) was used to design gene-specific primers used in qRT-PCR (**Table S1**). All reactions were carried out in hardshell 384-well PCR plates (supplied by Bio-Rad, Cat #HSP3805). ACTIN was used to normalize transcript levels. Absolute expression is calculated according to the 2^–ΔΔCt^ method (Pfaffl, 2001). The qRT-PCR reactions were done in triplicate from three biological replicates. Data are presented as the mean ± SEM.

### Immunoblotting and detection of GIGANTEA protein levels

For western blot analysis, the leaf samples of the GI::GI-TAP line were collected after 24 HPI at ZT-8 of the photoperiod. The GI::GI-TAP transgenic line has an endogenous GI promoter tagged with a Tandem Affinity Purification reporter (David et al., 2006). For the detection of the GI protein, the above-mentioned line was taken instead of Col-0, as GI protein levels at ZT-8 under SD conditions in Col-0 were barely detectable. The leaves were crushed in a mortar and pestle using liquid nitrogen. Total protein was extracted from 100 mg of leaf sample using the protocol according to Khaleda et al. (2017). and further used for quantification, SDS-PAGE, and western blotting. The quantification of the protein was done by amido black assay (Panigrahy, 2004). SDS denaturing 8% gels were prepared according to Panigrahy (2004). Nearly 25 µg of protein for each sample was loaded in acrylamide gels and run for approximately 2.5 h at 80 V until the protein ladder was completely separated followed by transferring onto PVDF membranes (Immobilon-P; EMD Millipore) for Western blotting using the semi-dry transfer system (Bio-Rad, model #**1703940**). The membrane was blotted against anti-GI monoclonal primary antibody (unpublished data) overnight at 4°C followed by incubation with secondary antibody anti-rabbit polyclonal HRP (supplied by Sigma Aldrich, Cat #A0545-1ML) for 2 h at room temperature. Detection was done using ECL substrate reagent (supplied by Bio-Rad, Cat #1705060). ImageJ software was used for densitometric quantification of Western blot and normalization with the loading controls against Coomassie brilliant blue staining. The experiment was reproduced thrice with biological replicates.

### Tissue fixation and histochemistry

Leaves were fixed with FAA (formalin–acetic–alcohol) substituted with methanol (45% methanol, 10% formaldehyde, 5% glacial acetic acid, and 40% distilled water) using a portable vacuum pump for 15–20 min. During the initial 15 min of fixation, the vacuum was drawn and released several times to properly preserve the cell organelles. Roots were incubated at 96°C for 1 min in 10% (w/v) KOH (Banhara et al., 2015; Mishra et al., 2018). Roots were stained with propidium iodide (PI) (1:1,000 v/v phosphate buffer). The plant tissues were incubated with 10% KOH for 30 min followed by 15 min incubation with alkaline H_2_O_2_. The tissues were then exposed to 2% HCl for 2 min. Trypan blue (0.1%) was used to visualize root cell wall morphology after fungal invasion. Fluorescent DNA stains such as DAPI (4′,6-diamidino-2-phenylindole) (1:1,000 v/v phosphate buffer) and acridine orange (0.1%) were used for staining the viable nuclei of stomatal guard cells.

### Light and confocal microscopy

A phase contrast microscope (Model #59556, Nikon, Japan) equipped with a digital sight DS-Fi1 camera (Nikon) was used for imaging. For confocal microscopy, a confocal microscope system (Leica SP8, Leica Application Suite*3.5.5.19976) operating on an inverted microscope (DMI8), equipped with 63× NA 1.3 glycerol immersion lenses (Leica, Wetzlar, Germany), was used for imaging. High-resolution Z stack images were recorded with fourfold line averaging and a 1.00-μm step size. Along with concurrent transmitted light images, fluorescence images were collected from 420–500, 500–550, and 620–650 nm using excitation at 405, 488, and 552 nm, respectively. All image series were captured with the same imaging conditions. Image intensities were quantified from image stacks in ImageJ (FIJI installation of version 1.47v, National Institute of Health, Bethesda, MD, USA). Images were prepared according to standard brightness and contrast settings in Photoshop (version CS4, Adobe Systems, San Jose, CA, USA) and ImageJ.

### Chlorophyll fluorescence assessment

The effect of *F. oxysporum* on photosynthetic electron transport in Col-0 and *gi-100* mutant lines of *A. thaliana* was estimated through OJIP transient analysis to analyze the photosystem activities under stress conditions. The chlorophyll fluorescence was measured in the top 3rd leaf using a Multifunction Plant Efficiency Analyzer (M-PEA, Hansatech Instruments Ltd, UK). White actinic light of 3,000 μmol photons m^−2^ s^−1^ was used for the fluorescence induction, which was recorded at wavelengths of approximately 685 nm. The energy fluxes were calculated as per the equations of the JIP-test using Image Lab Biolyzer HP3 software. The plant leaves were allowed to acclimatize to dark conditions for at least 30 min before the fluorescence signal was measured. The measurements were taken in the middle of the upper surface of fully developed leaves. The translated values of biophysical parameters of OJIP transients like the quantum yields (φPo, φRo, φDo, and ψEo), specific activities per reaction center (ABS/RC, DIo/RC, Tro/RC, Eto/RC, and REo/RC), performance indexes (PIs), and structure–function indexes (VJ, SM, and N) were plotted based on Strasser et al. (2004). Abbreviations of the formulas used are compiled in **Table S2**. Measurements were done in at least 10 different plants for each treatment with three leaves from each plant. The OJIP curve was created using the mean values in each condition.

### Leaf temperature analysis

The uninfected and infected plants 9 DPI were used for studying the plant temperature. The plants were kept in a dark room keeping the FLUKE Infrared Camera (Model No: Ti450 PRO) equidistant, i.e., 40 cm away from the plants. The thermal images were analyzed using the SMART VIEW software version 4.8. Twenty different parts of the plant showing the highest temperature were specifically selected using the software, and the temperatures were noted. Temperatures from a minimum of 10 plants from each biological replicate were analyzed and averaged to generate leaf temperature data.

### Phytohormone estimation

The plant leaf samples (~300 mg) were collected at 24 HPI using liquid nitrogen and grounded using tissue-lyser (Tissuelyser II manufactured by Retsch, Qiagen) and kept in a lyophilizer overnight. Defense phytohormones were estimated according to Vadassery et al. (2012) with some modifications. Briefly, lyophilized samples (~20 mg) were extracted in 1 ml of methanol containing 40 ng ml^−1^ of D6-JA (HPC Standards GmbH, Cunners dorf, Germany), 40 ng ml^−1^ of D4-SA, 40 ng ml^−1^ of D6-ABA, and 8 ng ml^−1^ of JA [13C6] isoleucine conjugate as internal standards. The homogenized samples were mixed in a shaker for 30 min and centrifuged at 14,000 rpm for 20 min at 4°C. The supernatants were collected, and the homogenates were again extracted with 500 µl of methanol and centrifuged. The supernatants were taken out and mixed with the previous extracts. All steps were performed at 4°C. The combined extracts were subjected to vacuum evaporation at ambient temperature and re-suspended in 500 µl of methanol. Samples were analyzed on an Exion LC (Sciex ^®^) UHPLC system using formic acid (0.05%) in water as mobile phase A and acetonitrile as mobile phase B. Separation was attained on a Zorbax Eclipse XDB-C 18 column (50 × 4.6 mm, 1.8 µm, Agilent) coupled with a triple Quadrupole-trap MS/MS system (Sciex 6500+) in negative ionization mode. The flow rate was 1 ml min^−1^ and the elution profile was as follows: 0–0.5 min: 5% B; 0.5–9.5 min: 5% to 42% B; 9.5–9.51 min: 42% to 100% B; 9.51–12 min: 100% B and 12.1–15 min: 5% B. Scheduled multiple-reaction monitoring (MRM) is used to precisely observe analyte parent ion → product ion with a detection window of 60 s according to Vadassery et al. (2012). Phytohormones were quantified relative to the signal of their corresponding internal standard’s concentrations.

### Statistical analysis

All the statistical analyses used in this study were performed using GraphPad Prism version 8.0.1. For the test of significant differences, two-way ANOVA with multiple comparison (Tukey and Sidak) and Student’s unpaired *t*-test were used to analyze grouped column graphs. Error bars represent the standard error of the mean (SEM).

## Results

### Severity of wilt disease due to *F. oxysporum* infection in Col-0 and *gi-100* plants

The report of GI’s involvement in susceptibility of *Arabidopsis* to the hemi-biotrophic fungus *Bipolaris sorokiniana* (Kundu and Sahu, 2021) prompted us to investigate GI function in plant defense against other pathogens, particularly the soilborne fungus *F. oxysporum*, which infects *Arabidopsis* at the vegetative stage. In this study, the probable role of GI in plant defense during the *Fusarium–Arabidopsis* association was investigated using a T-DNA insertion mutant of GI, i.e., *gi-100* (Huq et al., 2000).

At 9 DPI, the *gi-100* mutant plants were found to be more resistant to wilt disease infection with greener and expanded leaves as compared to the Col-0 plants. The effect of *F. oxysporum* on another *gi* mutant such as *gi-1* has been carried out and similar results were obtained. The plants showed disease resistivity upon *F. oxysporum* infection (**Figure 1B**), whereas the line overexpressing GI, i.e., 35S::GI, showed susceptibility to the wilt disease with less expanded and turgid leaves (**Figure 1B**). The disease severity percentage indicating the disease progression in the *gi-100* and *gi-1* mutants was significantly (*p* ≤ 0.05) slower than in the Col-0 plants, with the area of necrotic lesions (red arrows) in the Col-0 plants being significantly higher (nearly double) than in the *gi-100* mutants after 9 DPI (**Figures 1C, D**). Following 9 DPI, necrosis was also apparent on the leaves of the *gi-100*, *gi-1*, and Col-0 plants. Hence, all further analyses were carried out with *gi-100* plants. Trypan blue staining in leaf mesophyll cells of Col-0 (**Figure 1F**, upper panel) and *gi-100* (**Figure 1F**, lower panel) showed that non-viable cells are significantly higher in Col-0 than in *gi-100*. Here, we showed that the invasion of this root-borne fungus even through the stomata confirmed the infection through leaf. The fungal progression in Col-0 (**Figure 1G**, upper panel, right to left) and *gi-100* (**Figure 1G**, lower panel, right to left) roots from lateral root hair cells through the epidermal cells and tap root cells could visualize the gradual spread of the disease. These results clearly indicated that the progression of the disease was significantly less in *gi-100* compared to wild type.

### Effect of *F. oxysporum* infection on GIGANTEA transcript and protein levels

**Figure 2:**
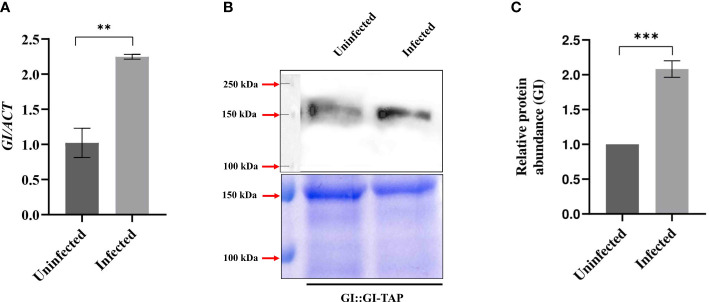
Transcript and protein levels of GIGANTEA after *F. oxysporum infection*. **(A)** Quantitative RT-PCR results showing *GI* transcript abundance in uninfected (0 HPI) and 24 HPI in Col-0 leaf samples. Transcript abundance in Col-0 uninfected at 24 HPI was taken as 1 to calculate the relative transcript level of *GI*. ACTIN was used to normalize transcript levels. **(B)** Immunoblot detection and **(C)** relative protein levels of GI in the uninfected and infected GI::GI-TAP leaf samples. **(B)** Bottom panels show SDS-PAGE stained with Coomassie brilliant blue (CBB). Top panel shows the PVDF membrane blotted against anti-GI monoclonal primary antibody raised in rabbit (unpublished data) and further incubated with secondary antibody anti-rabbit polyclonal HRP for 2 h at room temperature. Detection was done using ECL substrate. The corresponding loading control is shown by CBB-stained gel. **(C)** For relative protein levels, ImageJ software was used for densitometric quantification of Western blot and normalization with the loading controls against CBB staining. Calculations were done by taking protein abundance in uninfected conditions at 0 HPI as 100%. The qRT-PCR reactions were done in triplicate from three biological replicates. Data are presented as mean ± SE. To evaluate significant differences, Student’s unpaired *t*-test was used to compare between uninfected and infected Col-0 plants. **p < 0.01, ***p < 0.001.

As the absence of GI affected the severity of wilt disease, the transcript abundance and protein accumulation of GI before and after *F. oxysporum* infection at 24 HPI were studied to investigate the scenario at the molecular level. After 24 HPI, nearly a twofold rise in the level of GI transcript was observed in Col-0 leaf samples (**Figure 2A**). For protein levels, GI::GI-TAP was used to mimic the GI levels in Col-0 WT. In the immunoblot analysis of GI at 24 HPI following *F. oxysporum* infection, the protein abundance of GI was nearly double that of the uninfected samples (**Figures 2B, C**). These results confirmed that *F. oxysporum* infection causes an increase in GI protein, which may lead to susceptibility in *A. thaliana*.

### Effect of *Fusarium* infection on flowering time

**Figure 3:**
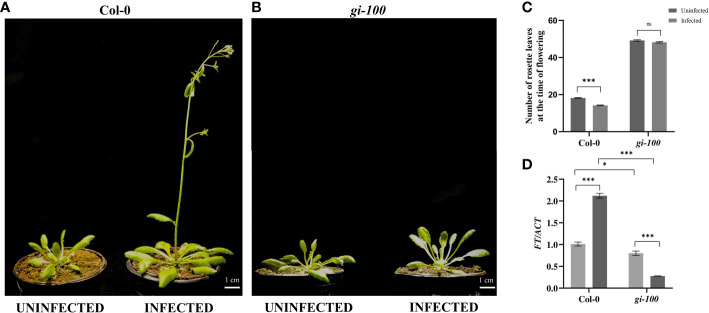
Analysis of flowering time phenotype in the *F. oxysporum*-infected Col-0 plants **(A)** Phenotype of 29-day mature Col-0 and **(B)** *gi-100* plants that were infected with *F. oxysporum* (9 DPI) and uninfected plant as control. **(C)** Number of rosette leaves in Col-0 and *gi-100* at the time of flowering. **(D)** Transcript expression analysis of *FT* gene using RT-qPCR after 24 HPI in Col-0 and *gi-100*. ACTIN was used to normalize transcript levels. The qRT-PCR reactions were done in triplicate from three biological replicates. Two-way analysis of variance (ANOVA) using Tukey’s multiple comparisons test was performed with the help of GraphPad Prism to test for significance among the dataset, *p < 0.05, ****p < 0.001. ns, Non-significant.

As any kind of biotic or abiotic stress alters the flowering and reproductive ability of a plant, the flowering phenotype of Col-0 plants was studied with or without *F. oxysporum* infection. Flowering time is reciprocally related with the total number of rosette leaves in *A. thaliana* (Mishra and Panigrahi, 2015). Moreover, GI has been demonstrated to control and regulate photoperiodic flowering in *A. thaliana* by upregulating the expression of *FT* gene (Wang et al., 2011). Col-0 plants flower with ~17 ± 2 leaves at the time of bolting (**Figures 3A–C**). Col-0 plants could not survive the infection with 70% lethality by 15 DPI. However, the Col-0 plants that could survive showed early flowering phenotype with ~12 ± 3 leaves at the time of bolting (**Figures 3A–C**). The uninfected *gi-100* plants show delayed flowering with 50 ± 2 rosette leaves at the time bolting. Even in the *gi-100* plants, *F. oxysporum* infection induced 30% lethality. However, in *gi-100*-infected plants, the flowering time was unaltered with similar number of rosette leaves as compared to their respective uninfected plants. These results indicated that during *F. oxysporum* infection, GI had minimal or no role in flowering time regulation. During plant–pathogen interactions, the expression of flowering regulatory genes including *FLOWERING LOCUS T* (*FT*) and *FLC* is known to be altered (Wang et al., 2011). Hence, the transcript expression of *FT* at 0 HPI and 24 HPI in Col-0 and *gi-100* was studied using the qRT-PCR approach. After infection, FT expression in Col-0 plants suddenly increased to >2-fold at 24 HPI. The expression of *FT* in *gi-100* was 0.79-fold lower than that of Col-0 plants under uninfected conditions (**Figure 3D**). At 24 HPI, the FT expression in *gi-100* plants was further reduced to ~2.9-fold. Together, these results indicated that the flowering time phenotype in *gi-100* might be regulated by several factors in addition to FT expression.

### Involvement of salicylic acid and jasmonic acid pathway during *F. oxysporum* infection in *gi-100* plants

**Figure 4:**
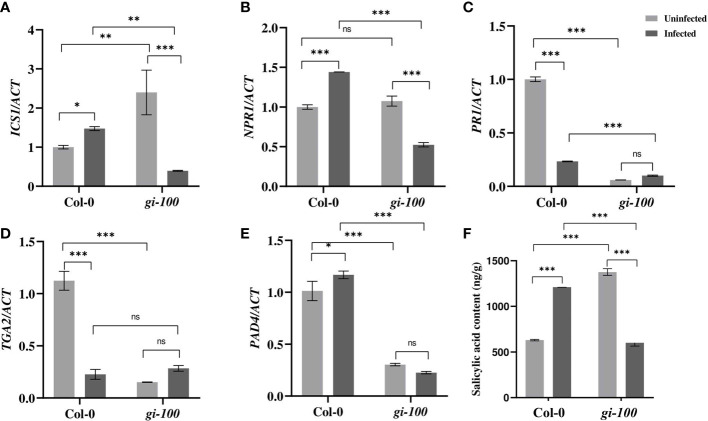
Involvement of the salicylic acid pathway during *F. oxysporum* infection in *gi-100* plant infection. **(A–E)** Transcript expression validation using RT-qPCR and **(F)** quantitative profiling of salicylic acid amount at 24 DPI by *F. oxysporum* were done in *gi-100* and Col-0 lines of *A thaliana.* **(A)** *ISOCHORISMATE SYNTHASE 1* (*ICS1*), **(B)** *NON-EXPRESSOR OF PATHOGENESIS-RELATED GENES 1* (*NPR1*), **(C)** *PATHOGEN RESPONSIVE GENE 1* (*PR1*), **(D)** *TGACG SEQUENCE-SPECIFIC BINDING PROTEIN 2* (*TGA2*), and **(E)** *PAD4*. ACTIN were used to normalize transcript levels. Determination of **(F)** salicylic acid (SA) amount. For SA quantification, D4-salicylic acid was taken as standard. Values are presented as mean ± SE. The qRT-PCR reactions were done in triplicate from three biological replicates. Two-way analysis of variance (ANOVA) using Sidak’s multiple comparisons test was performed with the help of GraphPad Prism to test for significance among the dataset, *p < 0.05. **p < 0.01, ***p < 0.001. ns, Non-significant.

To understand the molecular basis of resistance of *gi-100* plants to *F. oxysporum* infection, transcript expressions of genes of JA and SA signaling pathway were studied. JA and SA signaling are the primary respondents for conferring resistance to wilt disease during the biotrophic and necrotic phases of infection, respectively, with *F. oxysporum* (Edgar et al., 2006; Cole et al., 2014). Under uninfected conditions, the levels of *ISOCHORISMATE SYNTHASE 1* (*ICS1*) are low in Col-0 whereas it was ~2.3-fold induced in *gi* mutants. While there was a negligible increase in the transcript levels of *ICS1*, the SA biosynthesis gene in Col-0 plants after 24 HPI, it was drastically down (~6-fold) in the *gi-100* (**Figure 4A**) at the same time point. *NON-EXPRESSOR OF PATHOGENESIS-RELATED GENES 1* (*NPR1*) is involved in SA-mediated growth regulation by controlling cell division and expansion (Vanacker et al., 2001; Ushio et al., 2020; Wang et al., 2020; Li et al., 2022). The transcript expression of *NPR1* showed a ~0.6-fold increase in the Col-0 and a significant reduction of ~2-fold in the *gi-100* plant samples with infection (**Figure 4B**). *PATHOGEN RESPONSIVE GENE 1* (*PR1*) is a well-known pathogenesis-related protein family, and its accumulation is SA-dependent and linked to SAR (van Loon and van Strien, 1999; van Loon et al., 2006; Yang et al., 2018). In general, PR1 is associated with defense against biotrophs or hemi-biotrophs (Glazebrook, 2005; Yang et al., 2018). *PATHOGEN RESPONSIVE GENE 1* (*PR1*) transcript levels were decreased to ~4-fold in Col-0 after *F. oxysporum* infection (**Figure 4C**). To identify the downstream regulatory components that might be involved in controlling the expression of *PR1* upon *Fusarium* infection, the transcript accumulation of transcription factor *TGACG SEQUENCE-SPECIFIC BINDING PROTEIN 2* (*TGA2)* was investigated. The endogenous levels of *TGA2* in *gi-100* were found to be ~7.3-fold less than that of the Col-0 plants under uninfected conditions. However, 24 HPI, *TGA2* transcript expression levels reduced to 79% in the Col-0 samples. In contrast, in the *gi-100* samples, it increased by ~1.8-fold of its corresponding starting amount (**Figure 4D**). These results indicated an opposite trend of gene regulation of *TGA2* in the *gi-100* as compared to the Col-0. *PHYTOALEXIN DEFFICIENT 4* (*PAD4*) is an essential component in the modulation of phytoalexin biosynthesis in response to a range of bacterial and fungal diseases; hence, it is widely used to ameliorate plant resistance against pathogen infections (Cui et al., 2017). Similar to *TGA2*, the *PAD4* endogenous transcript levels were also significantly less in the *gi-100* plants before infection. While the *PAD4* transcript levels increase to ~1.15-fold in Col-0 at 24 HPI, it showed a minimal decrease in the *gi-100* leaves, nearly ~1-fold (**Figure 4E**). Thus, PAD4 regulation in *gi-100* also seemed to be in contrast to that in Col-0. These results indicated that SA pathway genes were contrastingly regulated in the *gi-100* samples post-infection as compared with their Col-0 counterparts. Moreover, the SA pathway was found to be positively regulated in *gi-100* after infection. Quantification of SA hormone in *gi-100* and Col-0 plants after 24 HPI was carried out to further investigate the basis of these results. The endogenous SA amount was found to be ~2.1-fold higher in the *gi-100* plants compared to the Col-0 plants before infection. However, after *F. oxysporum* infection, while the SA amounts increased significantly to 0.5-fold in Col-0 plants, it was decreased to nearly half, i.e., ~2.2-fold, in the *gi-100* plant samples (**Figure 4F**). These results further confirmed that GI positively regulates the SA pathway genes during response to *F. oxysporum* infection.

**Figure 5:**
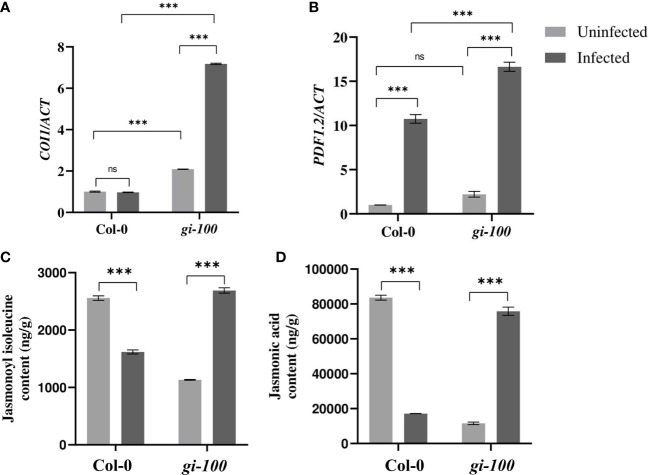
Involvement of the jasmonic acid pathway during *F. oxysporum* infection in *gi-100* plants. **(A, B)** Transcript expression validation using RT-qPCR and **(C, D)** quantitative profiling of jasmonic acid and jasmonyl-(L)-isoleucine amount at 24 DPI by *F. oxysporum* were done in *gi-100* and Col-0 leaf samples. **(A, B)** Relative transcript levels of **(A)** *CORONATINE INSENSITIVE 1* (*COI1*) and **(B)** *PLANT DEFENSIN1.2* (*PDF1.2*). ACTIN was used to normalize transcript levels. Determination of **(C)** jasmonic acid hormone and **(D)** jasmonyl-(L)-isoleucine JA-ile amounts. For JA and JA-ile quantification, D6-jasmonic acid and jasmonic acid-[13C6] isoleucine conjugate were taken as standard. The qRT-PCR reactions were done in triplicate from three biological replicates.Values are expressed as mean ± SD. Two-way analysis of variance (ANOVA) using Sidak’s multiple comparisons test was performed with the help of GraphPad Prism to test for significance among the dataset, ***p < 0.001. ns, Non-significant.

Having known about regulation of the SA pathway genes, JA pathway genes were also investigated. *CORONATINE INSENSITIVE 1* (*COI1*) act as a co-receptor in the perception of jasmonyl-(L)-isoleucine (JA-Ile) (Ruan et al., 2019). Though the amounts of JA were comparable between the Col-0 and *gi-100* plants before infection, its regulation was the opposite 24 HPI. There was not much difference in the expression of *COI1* in Col-0 samples; however, it showed a ~3.4-fold increase in *gi-100* after infection (**Figure 5A**). The *PLANT DEFENSIN1.2* (*PDF1.2*) is known as an important regulator of the JA signaling pathway (Ruan et al., 2019). Relative expression levels of *PDF1.2* were significantly increased in both the Col-0 and *gi-100* samples by ~10-fold and ~7-fold, respectively (**Figure 5B**), indicating a probable inhibition due to GI in the JA pathway. Further confirmation of the above results was done by quantification of JA and JA isoleucine (JA-ile) in *gi-100* and Col-0 leaf samples 24 HPI. After *F. oxysporum* infection, both the JA and the JA-ile plant hormone amounts showed opposite trends in Col-0 compared to the *gi-100* samples; i.e., while Col-0 samples showed ~4-fold and ~1.5-fold decrease, *gi-100* samples had ~6.5 and ~2.3-fold increase, respectively (**Figures 5C, D**). These results confirmed that GI negatively regulates JA pathway genes to confer resistance against *F. oxysporum* infection.

Moreover, it was also found that the ABA content was also ~1.6-fold higher in *gi-100* plants than the minimal change observed in the Col-0 plants 24 HPI (**Figure S2A**). Oxylipins are a new class of signals that enhance virulence in plants under biotic stress tolerance (Reverberi et al., 2012). The endogenous oxylipin amount was less in the *gi-100* samples before infection, but increased 24 HPI in both the Col-0 and *gi-100* plant samples irrespective of the genotype (**Figure S2B**). This result suggested that *gi-100* mutants were highly resistant to *F. oxysporum* infection, probably due to lower oxylipin synthesis in *gi-100* mutants. The increase in oxylipin content in Col-0 plants suggests that having GI in the host increased vulnerability by increasing oxylipin content in plants following *F. oxysporum* infection.

### Comparative anatomy of the effect of *F. oxysporum* infection on root cell structure in Col-0 and *gi-100* plants

**Figure 6:**
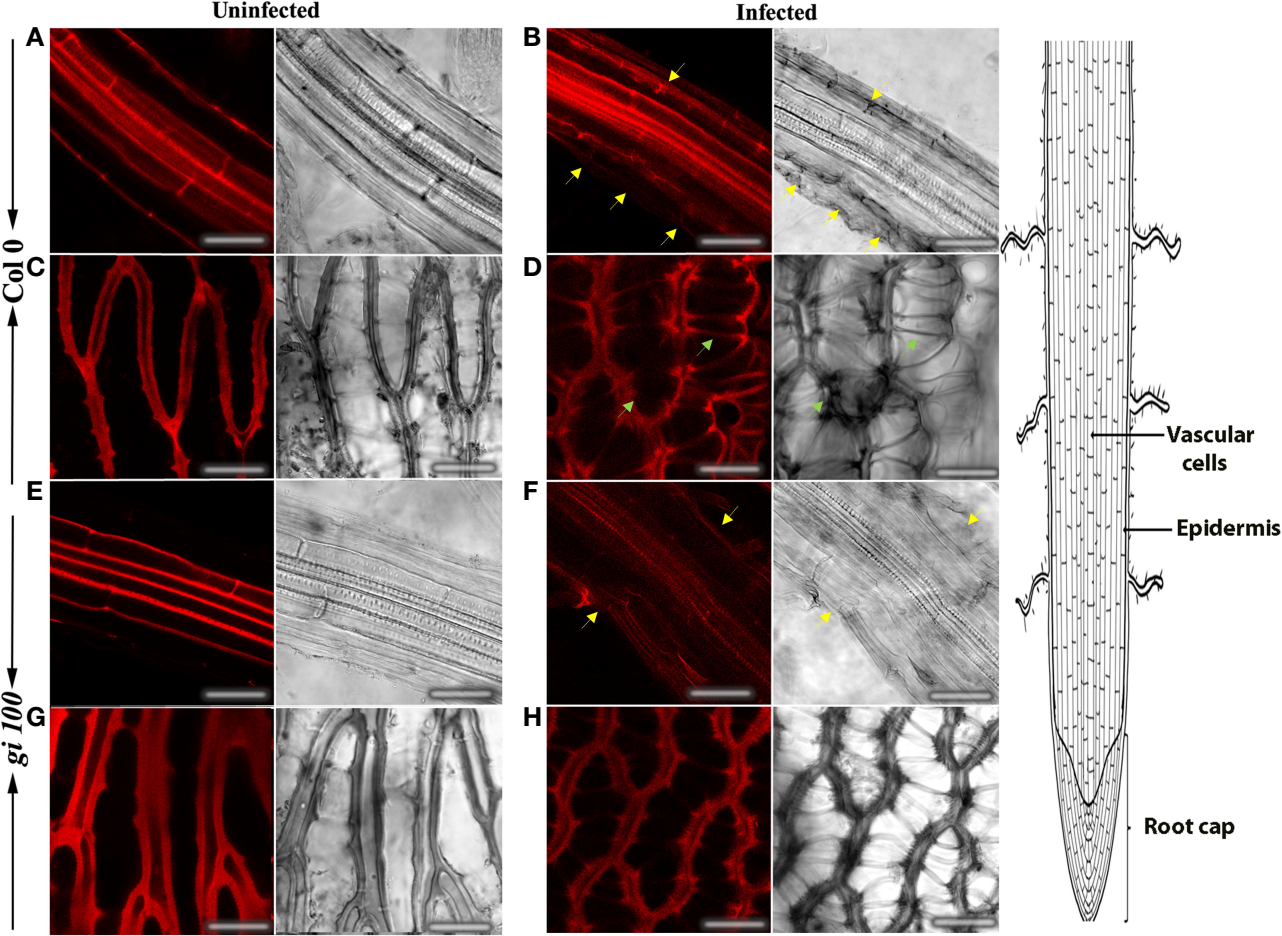
Effect of *F. oxysporum* infection on root cells in *Arabidopsis thaliana* using propidium iodide. Concurrent confocal fluorescence and transmitted light images of uninfected and infected roots from Col-0 **(A–D)** and gi-100 **(E–H)** at 9-DPI stained with propidium iodide. Epidermal cell wall showed structural deformities in infected Col-0 **(B)** and gi-100 **(F)** (yellow arrows). Columella cells showed structural irregularities in cell shape of infected Col-0 (green arrows in **D**). Scale bar, 100 μm.

To further investigate the extent of *F. oxysporum* infection under our experimental regime and to determine the changes caused by the pathogen infection and its colonization strategy, confocal microscopy was carried out in the root cells of Col-0 and *gi-100* at 9 DPI. Roots inoculated with *F. oxysporum* strain revealed several deformities in the epidermal cell walls in both Col-0 and *gi-100* both in confocal microscopy (**Figures 6A, B, E, F**) and after trypan blue staining (**Figure S2**). The damage in the epidermal root cells of *gi* mutants was least compared to Col-0 (**Figures 6B, F)**. The root cap cells (columella cells, Wachsman et al., 2015) showed structural irregularities in cell shape of infected root tissues in both genotypes, which were fewer in *gi-100* as compared to Col-0 (**Figures 6C, D**, **G, H**). These results indicated that the damage in the root cap cells induced by *F. oxysporum* infection was less in *gi-100* plants than in Col-0 plants. This structural disorganization could be due to the mycotoxins produced by the fungus after infection (Perincherry et al., 2019).

### Effect of *F. oxysporum* infection on stomatal density and integrity in Col-0 and *gi-100* plants

**Figure 7:**
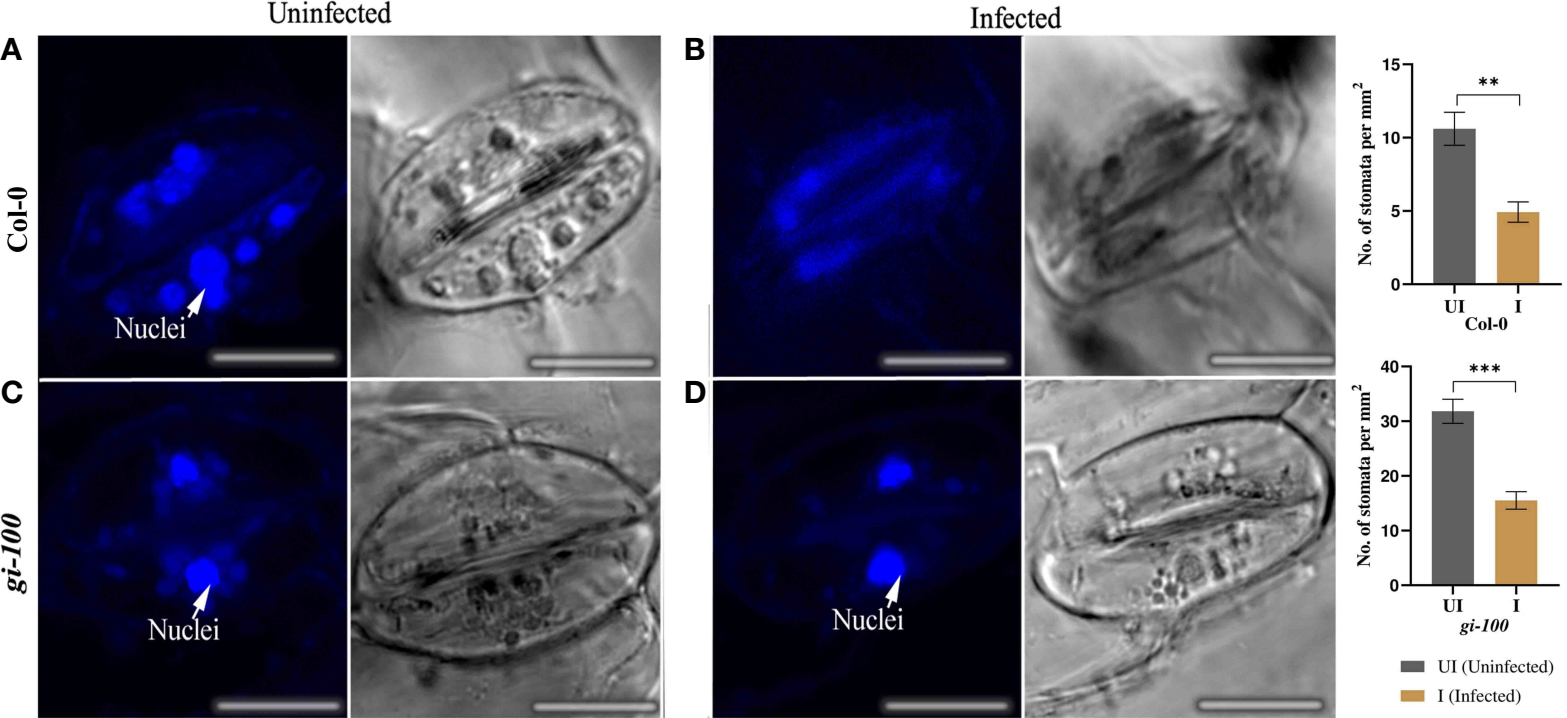
Effect of *F. oxysporum* infection on stomatal density and integrity in Col-0 and *gi-100* plants. Concurrent confocal fluorescence and transmitted light images of uninfected **(A, C)** and infected **(B, D)** *Arabidopsis* leaves at 9 DPI and labeled with DAPI stain for visualizing the nuclei. Nuclei are marked with arrows. Number of stomata was counted from the images taken during microscopy and calculated per unit area. Scale bar, 100 µm. Values in the graphs are expressed as mean ± SE. Two-way analysis of variance (ANOVA) using Sidak’s multiple comparisons test was performed with the help of GraphPad Prism to test for significance among the dataset,**p < 0.01, ***p < 0.001.

Number of stomata was higher in the *gi-100* than the Col-0 uninfected leaf cells. However, the stomatal density was reduced in cases of both Col-0 and *gi-100* upon *F. oxysporum* infection (**Figure 7**). The structural changes in the cell wall of stomata guard cells and disintegration of nuclei were observed in the guard cell of Col-0-infected leaves as an indication of cell death (**Figure 7**). Nevertheless, intact guard cell with nuclei could be visualized in the *gi-100*-infected leaves (**Figure 7**). These observations in the stomatal guard cells were reclaimed with acridine orange staining, which stains the cell wall of stomatal guard cells (**Figure S3**).

### Effect of *F. oxysporum* infection on chlorophyll A fluorescence and photosynthetic electron transport

**Figure 8:**
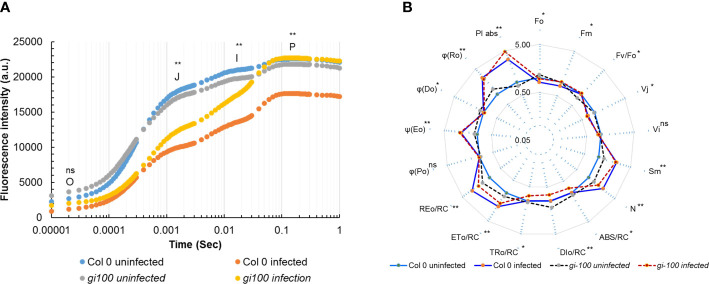
Effect of *F. oxysporum* on Chl a fluorescence. Col-0 WT and *gi-100* leaves were incubated at 24 DPI with the Multifunction Plant Efficiency Analyzer (M-PEA) (Hansatech Instruments Ltd, UK) for obtaining chlorophyll. **(A)** Fluorescence intensity on OJIP transient curve. **(B)** Spider plot for Col-0 and *gi-100* lines of *A. thaliana* plants 24 DPI with *F. oxysporum* with their respective control. Values are expressed as mean ± SD. Two-way analysis of variance (ANOVA) using Sidak’s multiple comparisons test was performed with the help of GraphPad Prism. *p < 0.05. **p < 0.01, ns, Non-significant.

To attain a deeper understanding of photosynthetic parameters, a Chl *a* fluorescent transient curve was generated from Col-0 and *gi-100* lines, 24 HPI with *F. oxysporum*. In the J-I phase, a significant decrease was observed in infected Col-0 and *gi-100* lines where Col-0 showed minimum fluorescent signals, which was also continued in the I-P phase. However, *gi-100* showed a steady increase of *Chl a* fluorescence from J-P and reached the level of uninfected plants (**Figure 8**). Infection of plants with *F. oxysporum* significantly decreased Chl F levels at J-step (Vj) in Col-0 and *gi-100* lines as compared to their respective control (untreated) but was slightly higher in *gi-100* than in Col-0 plants. A significant increase of Chl F in I (Vi) with the infected *gi-100* plants was recorded as compared to Col-0-infected plants at P (Vp) phases (**Figure 8B**).

### Leaf internal temperature after *F. oxysporum* infection

**Figure 9:**
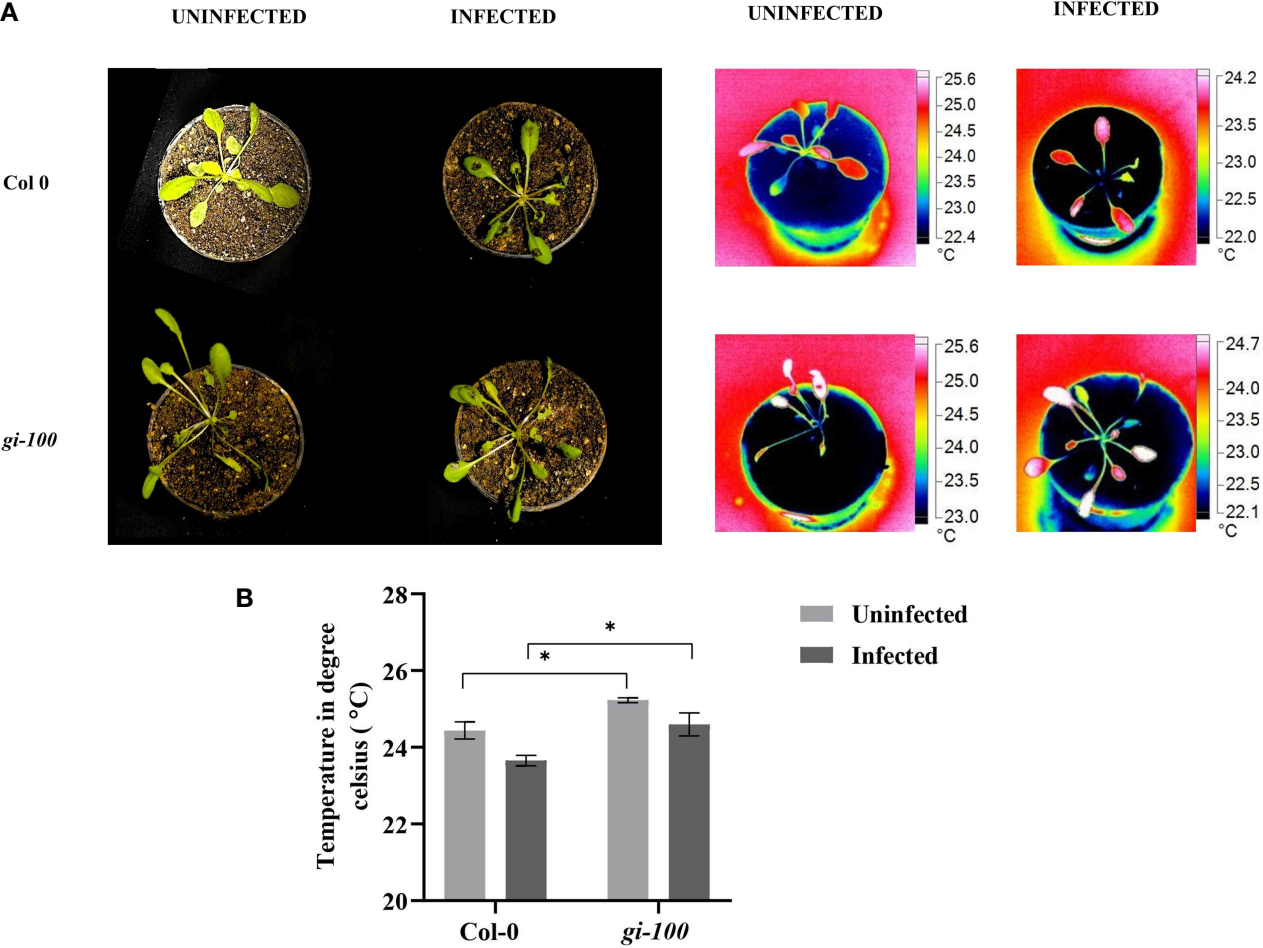
Leaf internal temperature analysis in Col-0 and *gi-100*. **(A)** Representative images of Col-0 and *gi-100* plants without and with *F. oxysporum* infection at 9 DPI. The pictures were taken with a FLUKE infrared camera and analyzed using the SMART VIEW software as described in *Materials and methods*. **(B)** Leaf internal temperature from a minimum of at least 10 plants of Col-0 and *gi-100* was taken. Leaf surface showing highest temperatures were selectively chosen. Values are expressed as mean ± SD. Two-way analysis of variance (ANOVA) using Sidak’s multiple comparisons test was performed with the help of GraphPad Prism *p < 0.05.

As phytochrome B has been shown to act as a thermosensor (Legris et al., 2016) and GI is involved in the phytochrome signaling responses (Huq et al., 2000), the involvement of GI in controlling a plant’s internal temperature was investigated and compared among the infected and uninfected plants to determine the effect of *F. oxysporum* infection (**Figure 9**). The average leaf temperature of *gi-100* plants was ~1.1°C (*p* < 0.05) higher than that of Col-0 plants (**Figure 9B**). The average leaf temperature of both Col-0 and *gi-100* plants decreased insignificantly due to *F. oxysporum* (**Figure 9A**). This result indicated that GI probably has a role in maintaining the lower internal temperature of Col-0 plants.

## Discussion

GI is ubiquitously present in plants at all stages of development (Mishra and Panigrahi, 2015), suggesting that it is involved in various physiological activities including flowering time regulation (Park et al., 1999), light signaling (Martin-Tryon et al., 2007), starch accumulation (Eimert et al., 1995), circadian clock control (Park et al., 1999), transpiration (Sothern et al., 2002; Tseng et al., 2004), chlorophyll accumulation (Kurepa et al., 1998), and miRNA processing (Jung et al., 2007). In plants, GI has also been demonstrated to regulate abiotic stressors such as drought (Riboni et al., 2013), cold (Cao et al., 2005; Fornara et al., 2015), salt (Kim et al., 2013), and oxidative stress (Kurepa et al., 1998), although its involvement in pathogen attack is yet unknown. Recent research reveals that during a fungal pathogen infection, *Bipolaris sorokiniana*, GI targets the SA pathway in *Arabidopsis* and wheat, making the plant more susceptible to the disease (Kundu and Sahu, 2021). GI, a photoperiodic pathway regulator, also improves susceptibility to *F. oxysporum*, according to RNA-seq data analysis (Lyons et al., 2015a; Lyons et al., 2015b). The *Atgi-1* and *Atgi-2* mutants are resistant to *F. oxysporum* infection (Lyons et al., 2015a; Lyons et al., 2015b); however, the mechanism behind this is still unknown.

In this study, we have demonstrated the causative mechanism of involvement of GI in *F. oxysporum*-induced wilt disease. Progression of the disease was confirmed by the visible disease symptoms on the leaves at 6 DPI, which is also supported by previous findings (Lyons et al., 2015a). Our study claimed that though the mode of infection in *F. oxysporum* is commonly known to be root borne (Jones and Dangl, 2006), it can also induce pathogenicity through leaf infections under laboratory conditions. The integrative growth approach of the initial 15 days of LD conditions followed by the rest of the study under SD conditions was done to attain a synchronized growth morphology with comparable number of rosette leaves in all the three genotypes used in the study. Following *F. oxysporum* inoculation, we noticed an increase in the expression of GI and FT in Col-0. Our findings showed that *F. oxysporum* infection causes early flowering in Col-0 plants and unaltered flowering in *gi-100* plants. These results suggested that GI may not play a primary role in flowering time regulation during infection. Despite the decrease in FT expression to nearly half at 24 HPI, the flowering time was unaltered in *gi-100* plants. This observation indicated that the flowering time could be regulated by other factors in *gi-100* plants and warrants the study of the expression of FT and other related factors at the time of bolting after pathogen infection. Nevertheless, our observation of decrease in FT expression in *gi-100* uninfected plants is supported by a similar observation in *gi-1* (Kim et al., 2013).

We hypothesized that the GI-mediated disease mechanism is blue light-dependent since GI is related to blue light receptors such as ZEITLUPE (ZTL), FLAVIN-BINDING, KELCH REPEAT, F-BOX 1 (FKF1), and LOV KELCH PROTEIN 2 (LKP2) for its function (Cha et al., 2017). G-BOX BINDING FACTOR 1 (GBF1), a blue light-induced transcription factor, has recently been found to have a positive effect on disease tolerance in recent studies (Giri et al., 2017). To further study the mechanism by which GI induces susceptibility during *F. oxysporum* infection, we have checked the accumulation of SA, JA, and ABA phytohormones. Our finding suggests that during *F. oxysporum* infection, GI inhibits the JA pathway and ABA accumulation while stimulating the SA pathway. DELLA protein regulates plant pathogen defense in *Arabidopsis*, according to recent research. Furthermore, the GI protein has been demonstrated to regulate gibberellic acid signaling by stabilizing the DELLA protein (Nohales and Kay, 2019). More studies can shed light on the GI–DELLA interaction and its involvement in plant pathogen response.

*F. oxysporum*, being a hemi-biotrophic pathogen, expands its hyphae in the host cells during the initial days of infection and subsequently produces mycotoxins, which kill the host plant (Lagopodi et al., 2002). Hence, any kind of macroscopic lesions or aberrations is not visible on the plant during the initial phase of infection. Concurrently, we did not observe any phenotypic changes in *A. thaliana* during the initial phase of infection, i.e., 1 to 4 DPI. However, at 9 DPI, the Col-0 WT plants displayed a susceptible phenotype to the disease with at least 60% of leaf area under necrosis. Since the fungal hyphae are known to invade the plant’s root system through the root hairs (Lagopodi et al., 2002; Banhara et al., 2015) and block the vascular bundles of the plant, we investigated the root cells of the infected and uninfected plants using confocal microscopy. PI followed by trypan blue stain was used to stain the plant cell walls to determine the extent of fungal infection in the root cells. Noticeable structural changes could be observed in the infected epidermal cells as compared with the uninfected epidermal root cells in both Col-0 and *gi-100* plants at 9 DPI. The epidermal cells of Col-0 were found to be ruptured to a greater extent than *gi* mutants. This could be due to the mycotoxins produced by the pathogen. However, staining the fungal hyphae with trypan blue at a later stage of infection could not be done due to the ruptured cells and overlapping of vascular root cells on the root cap cells. Moreover, plugging in the vascular bundles was found specifically in the Col-0 plants, which was probably the primary reason for the Col-0 plants’ susceptibility to the disease. The nuclei in stomatal guard cells were also observed to be disintegrated in Col-0, indicating cell death, whereas the nuclei in guard cells were intact even after infection, confirming the resistivity of the plant towards *F. oxysporum* infection.

**Figure 10:**
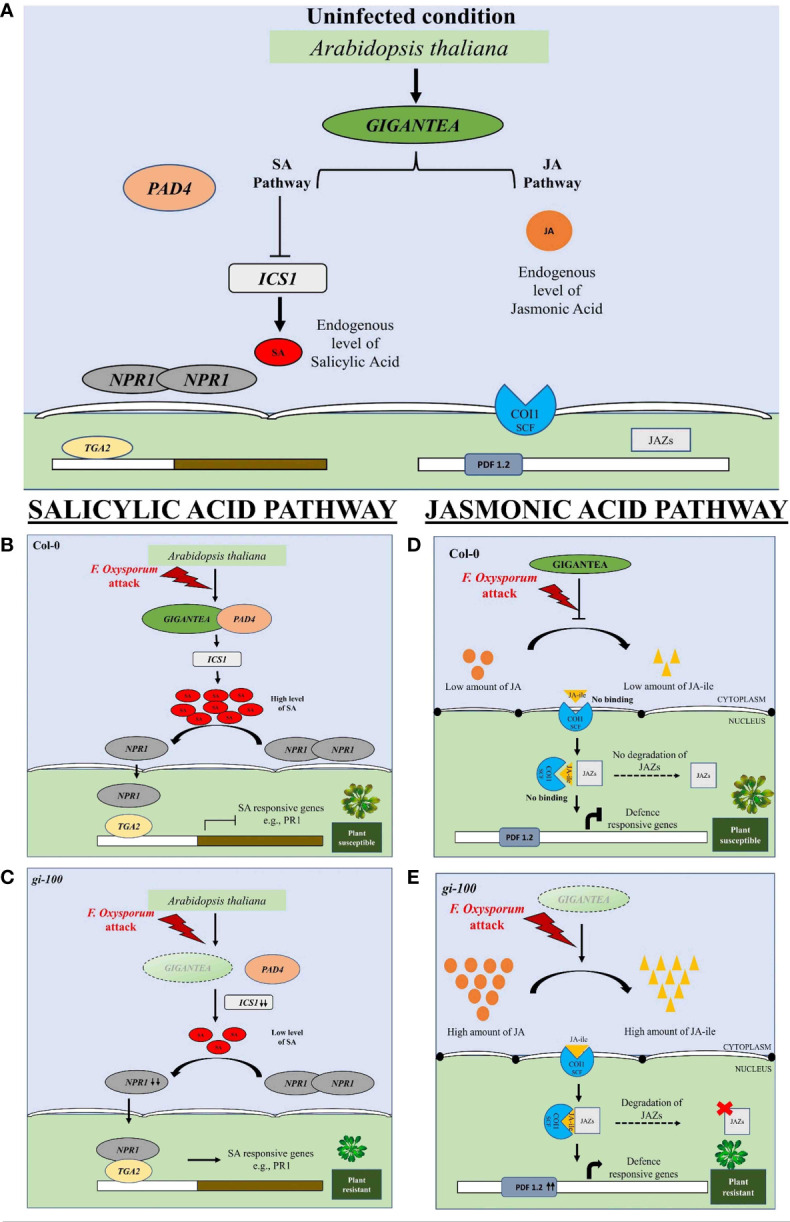
Diagrammatic representation of involvement of GIGENTEA, jasmonic acid, and salicylic acid pathway genes for eliciting pathogen response to *F. oxysporum* infection in *Arabidopsis thaliana*. **(A)** Under uninfected condition, jasmonic and salicylic acid levels and GI are at endogenous levels. The levels of ICS1 are low due to its inhibition. The dimerized form of NPR1 is unable to move into the nucleus and bind to any TF for defense response. Similarly, the JA pathway is also uninduced. **(B)** Salicylic acid pathway: When *F. oxysporum* infects wild-type Col-0 plants, increased GIGANTEA (GI) accumulation results in GI binding to PAD4, followed by increased SA accumulation. However, when infected, NPR1 is unable to bind to the transcription factor TGA2, limiting the expression of defense-related genes, i.e., PR1. **(D)** In the absence of GI, the SA pathway is reversed upon *F. oxysporum* infection, making the plant more resistant to the disease. **(C)** Jasmonic acid pathway: Increased GI expression and accumulation, on the other hand, results in less JA-ile in Col-0 (active form). Subsequently, there is a reduction in the transcript level of *COI1*, which does not bind to JA-ile and thus cannot form a complex with JAZs. JAZs functions as a repressor, inhibiting PDF 1.2. This eventually makes the plant vulnerable to *Fusarium* infection. **(E)** On the contrary, in the absence of GI, the accumulation of JA-ile is high and it provides resistance to the plant upon *F. oxysporum* infection.

Phytochrome B was recently reported to act as a thermosensor due to its temperature sensing property in plants (Legris et al., 2016). We presume the interplay of Phy-B signaling and involvement of PHYTOCHROME INTERACTING FACTORs (PIF-4, PIF-7) module in this process. GI is known to control phytochrome signaling during hypocotyl elongation in *A. thaliana* (Huq et al., 2000). This prompted us to investigate the plant’s internal temperature during *F. oxysporum* infection in the presence and absence of GI. The noble finding of higher temperature in the *gi-100* plants regardless of *F. oxysporum* infection indicated that GI plays a role in controlling plants’ internal temperature. A similar report of weakened plant immune defense during a rise in temperature has been shown (Bosman, 2022; Kim et al., 2022). Hence, integrating both results, it could be suggested that the influence of light and temperature on the host–pathogen association in plants may be mediated by GI. Based on the findings of the present study, we propose a model in which GI acts as a negative regulator of defense signaling in *Arabidopsis* and can contribute to the susceptibility to wilt disease (**Figure 10**). Explicitly, *Fusarium* infection in Col-0 induces GI expression and accumulation, resulting in the reduced endogenous JA amount followed by the reduced JA-ile (active form) amount. Consequently, a decrease in the accumulation of *COI1* transcript level, which is unable to bind to JA-ile, forms a complex with JAZs. The JAZs act as a repressor to inhibit *PDF 1.2.* This eventually leads the plant to become susceptible to *Fusarium* infection. On the other hand, the susceptibility of Col-0 to the vascular wilt disease caused by the *F. oxysporum* was found to be positively regulated by the SA pathway. Therefore, in *gi* mutants, the SA content as well as the ICS1 transcript levels are high under uninfected conditions. After the plant was attacked by the pathogen, there was an increased transcript expression of PAD4, which corroborated with the fact that GI binds with the PAD4 intronic region upon pathogen infection (Singh, 2022). The level of *ICS1* transcript increased, as a result of which the amount of SA metabolites also increased in the plant. The *NPR1* transcript levels were also elevated after the pathogen infection. However, the transcript levels of *TGA2*, a transcription factor, decreased in the presence of GI in Col-0 after infection. TGA bZIP factors can function as either repressors or activators, or both. In the absence of SA stimulation, TGA2 binds to the PR-1 promoter and inhibits transcription. NPR1 binds to TGA2 at the promoter in SA-stimulated cells, masking TGA2’s repressor domain. In an SA-dependent manner, the protein complex activates transcription. TGA2 thus acts as a repressor or a coactivator in this context (Rochon et al., 2006). However, in our case, the transcription level of TGA2 was low after pathogen infection, as result of which the complex formation of NPR1 and TGA2 is hindered. This might be due to the fact that some fungal toxins are secreted by the pathogen and prevent the NPR1 to mask the TGA’s repressor domain. It was also observed that the *PR1* transcript (SA defense responsive gene) significantly decreased in Col-0 WT. In contrast, *gi* mutation causes the release of this positive suppression of pathogen-induced SA signaling, resulting in increased resistance of *gi 100* plants. As a result, we conclude that the GI module promotes susceptibility to *F. oxysporum* infection by promoting the SA pathway and inhibiting the JA pathway in *A. thaliana*.

## Conclusion

The present study significantly contributes to understanding the involvement of the circadian clock component GIGENTEA in controlling biotic stress response during the pathogen infection by *F. oxysporum* in *A. thaliana*. This is one of the few reports that explores the molecular mechanisms and involvement of GI for biotic stress tolerance (specifically against *F. oxysporum* infection, which causes vascular wilt disease in Columbia-0 wild-type plants). Disease resistance in *gi-100* for the said disease is positively regulated by modulating JA pathway genes [i.e., *CORONATINE INSENSITIVE 1* (*COI1*) and *PLANT DEFENSIN 1.2* (*PDF1.2*)]. In addition, our report also reveals the involvement of ABA, SA pathways genes, and oxylipins in this response. Though GI promotes early flowering under controlled conditions, our report also showed that it is not involved in flowering time regulation during *F. oxysporum* infection. This is the first study to report that GI is involved in maintaining lower temperatures of plants, as *gi-100* plants maintained a higher temperature irrespective of the pathogen infection. The findings of this study can be useful in building stronger strategies for improving crop yield in fields in case of *F. oxysporum* infection by manipulating the genes of JA and salicylic pathways.

## Data availability statement

The original contributions presented in the study are included in the article/**Supplementary Material**. Further inquiries can be directed to the corresponding author.

## Author contributions

The experiments were conceived and designed by KP and AP. The experiments were carried out and data were analyzed by AP and AK. The pathogen was grown, samples were collected, and the plants were infected by AP, AK, AB, SD, GM, and AD. The manuscript was written by AP, MP, and KP, and the figures were finalised by AP, MP and KP. All authors contributed to the article and approved the submitted version.

## Supplementary material

Supplementary Figure 1 Effect of *F. oxysporum* infection on root cells morphology. Longitudinal root tissue sections from uninfected and 9-DPI were stained with trypan blue for epidermal cell wall morphology. Epidermal cell wall of vascular cells showed structural changes in infected Col-0 and *gi-100* root cells. Arrow pointing to ruptured epidermis in B, D. Scale bar 100 µm.

Supplementary Figure 2 Determination of Abscisic acid and Oxylipins after *F. oxysporum* in Col 0 and *gi-100* plants. Determination of endogenous ABA **(A)** and oxylipins **(B)** amount at 9-DPI by *F. oxysporum* in *gi-100* as well as Col-0 leaf samples. Data points represent mean ± SD. Two-way analysis of variance (ANOVA) using Sidak’s multiple comparisons test was performed with the help of GraphPad prism *p<0.05. **p<0.01, ***p<0.001.

Supplementary Figure 3 Effect of *F. oxysporum* infection on stomatal number and integrity in *Col-0 and gi-100* plants. Concurrent confocal fluorescence and transmitted light images of uninfected and infected *Arabidopsis* leaves were taken which were labelled with Acridine orange. Arrow points to the nuclei of guard cells in uninfected Col-0 and infected *gi-100* lines. Scale bar 100 µm.
