# Supplementary figures and images for: GIGANTEA supresses wilt disease resistance by down-regulating the jasmonate signaling in Arabidopsis thaliana
Source: Front Plant Sci. 2023 Mar 9;14:1091644. doi: 10.3389/fpls.2023.1091644 (PMC10034405; doi:10.3389/fpls.2023.1091644)

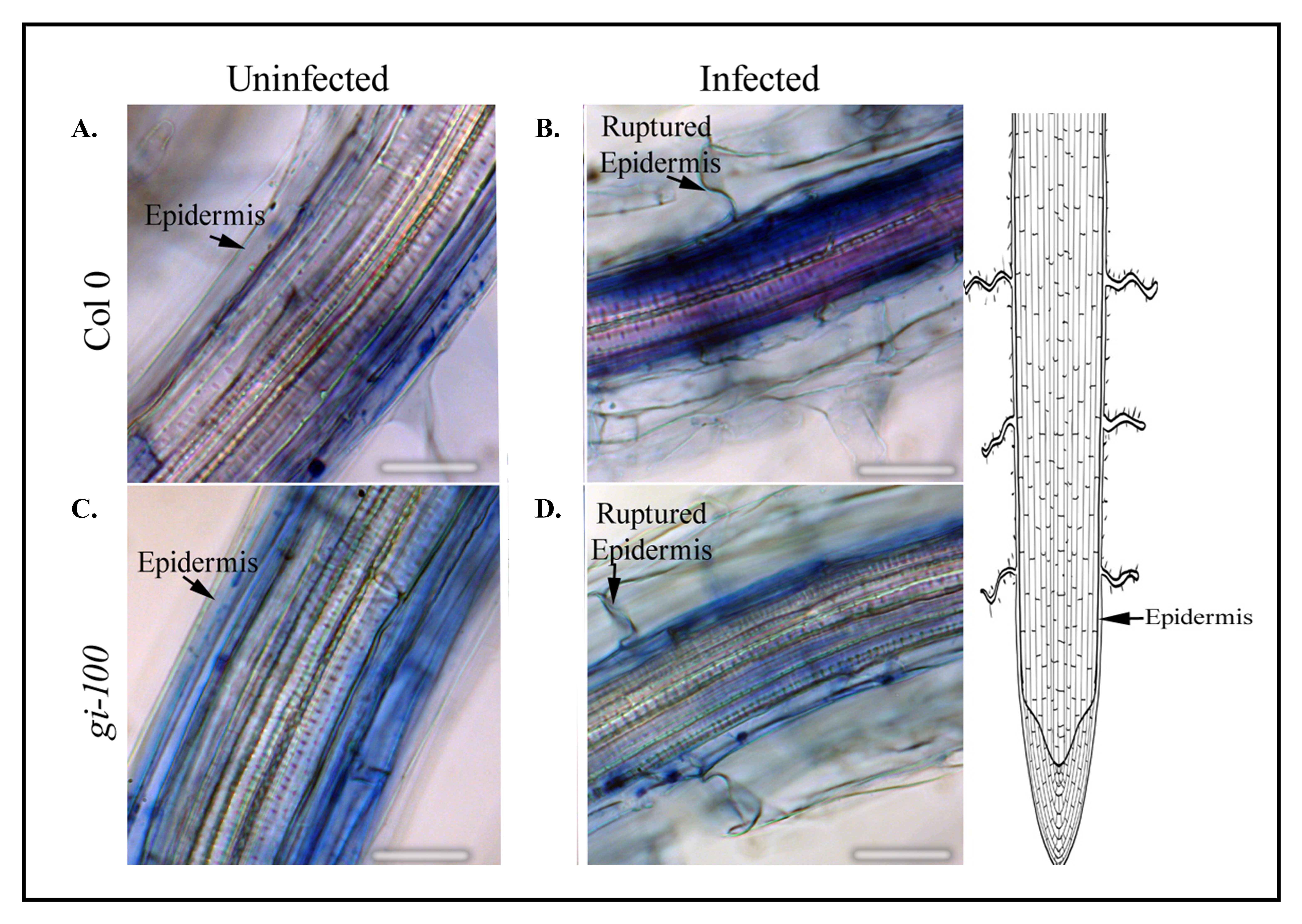

Supplement: Supplementary Figure 1 — Effect of F. oxysporum infection on root cells morphology. Longitudinal root tissue sections from uninfected and 9-DPI were stained with trypan blue for epidermal cell wall morphology. Epidermal cell wall of vascular cells showed structural changes in infected Col-0 and gi-100 root cells. Arrow pointing to ruptured epidermis in B, D. Scale bar 100 µm. [file Image_1.jpeg]

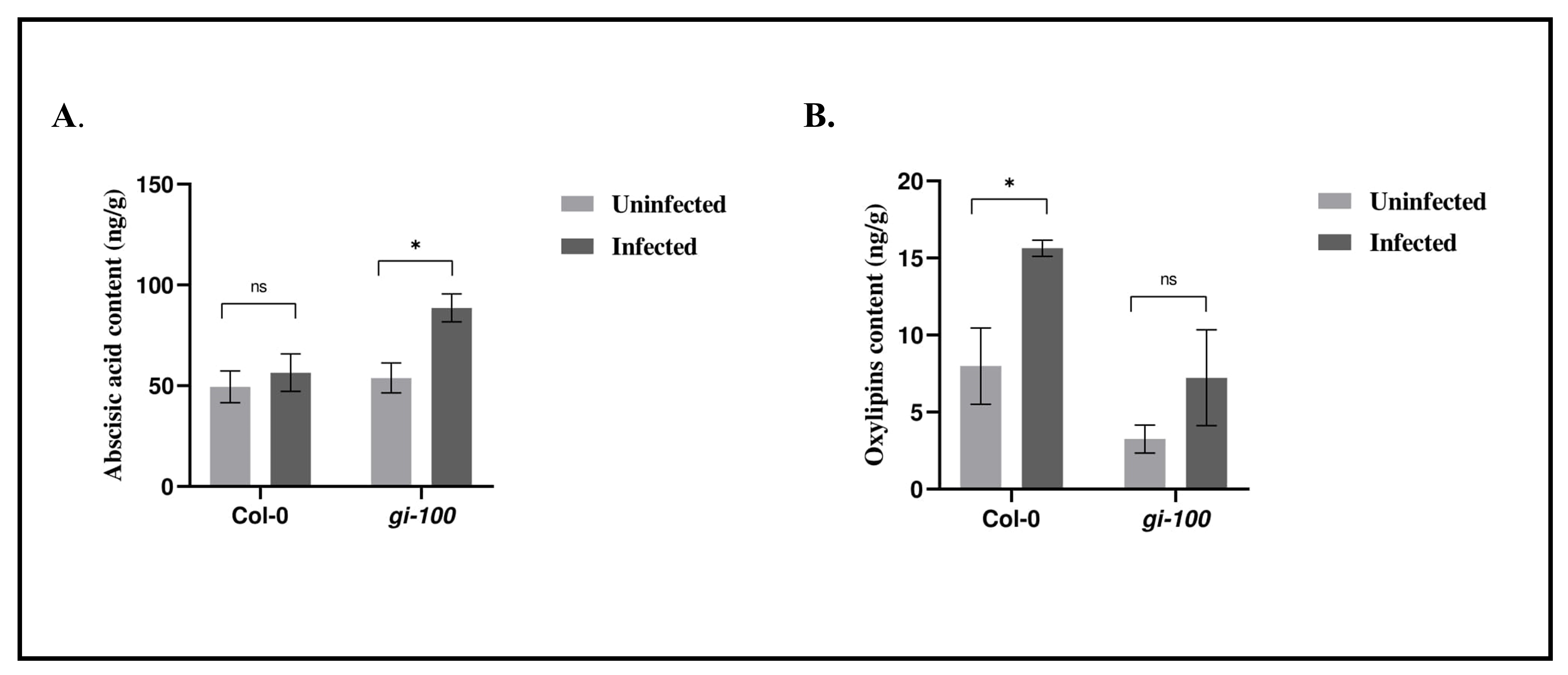

Supplement: Supplementary Figure 2 — Determination of Abscisic acid and Oxylipins after F. oxysporum in Col 0 and gi-100 plants. Determination of endogenous ABA (A) and oxylipins (B) amount at 9-DPI by F. oxysporum in gi-100 as well as Col-0 leaf samples. Data points represent mean ± SD. Two-way analysis of variance (ANOVA) using Sidak’s multiple comparisons test was performed with the help of GraphPad prism *p<0.05. **p<0.01, ***p<0.001. [file Image_2.jpeg]

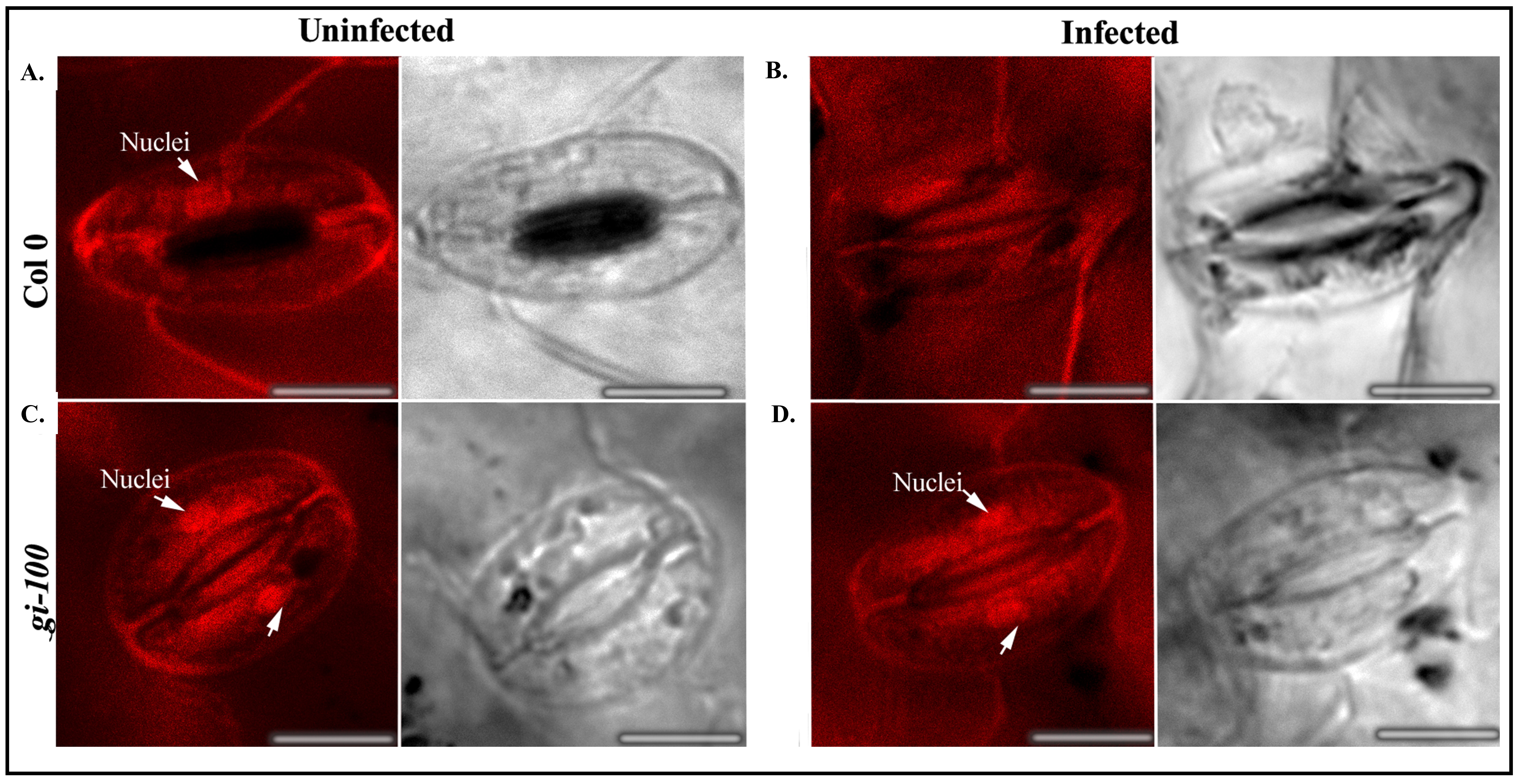

Supplement: Supplementary Figure 3 — Effect of F. oxysporum infection on stomatal number and integrity in Col-0 and gi-100 plants. Concurrent confocal fluorescence and transmitted light images of uninfected and infected Arabidopsis leaves were taken which were labelled with Acridine orange. Arrow points to the nuclei of guard cells in uninfected Col-0 and infected gi-100 lines. Scale bar 100 µm. [file Image_3.jpeg]
