# Supplementary material for: GIGANTEA supresses wilt disease resistance by down-regulating the jasmonate signaling in Arabidopsis thaliana
Source: Front Plant Sci. 2023 Mar 9;14:1091644. doi: 10.3389/fpls.2023.1091644 (PMC10034405; doi:10.3389/fpls.2023.1091644)
Supplement: Supplementary file 4 [file Table_1.docx]

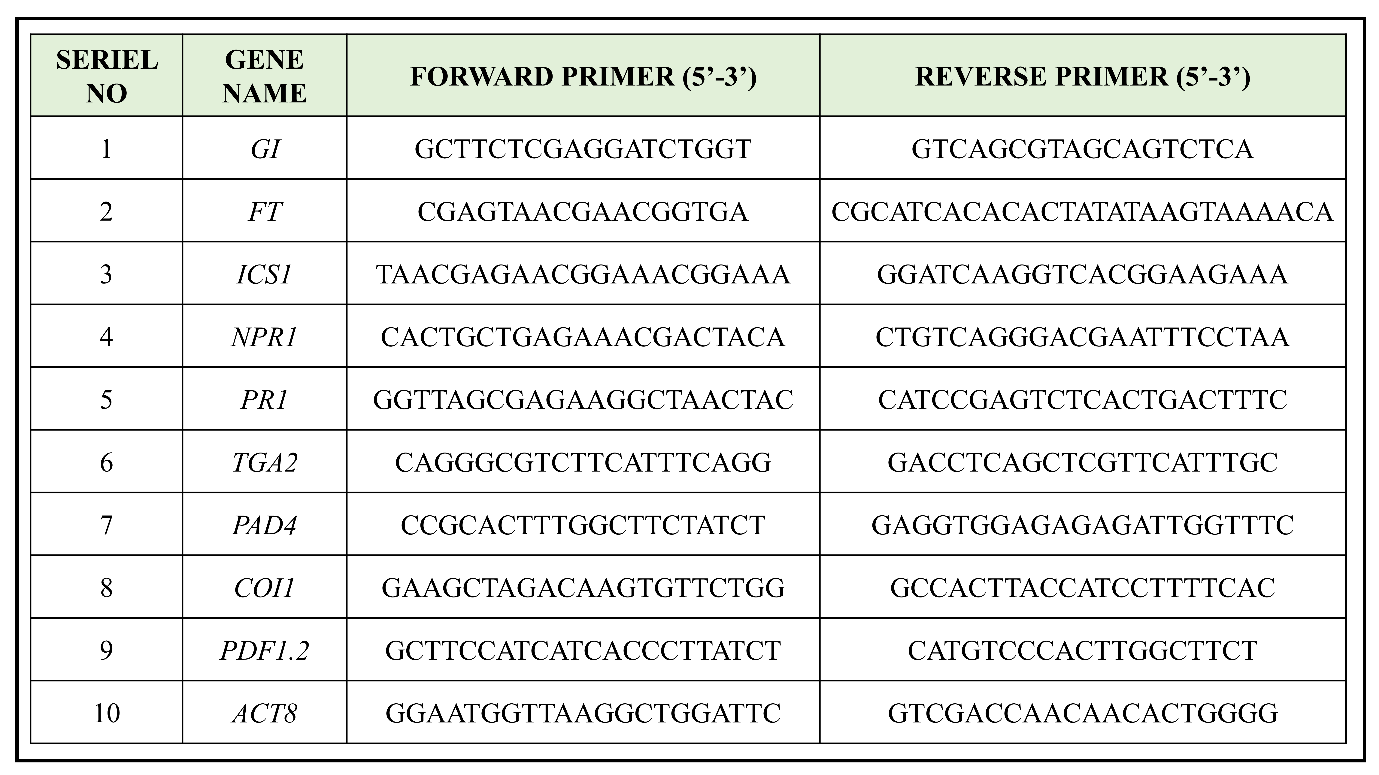


**Table S1. List of primers used for q-PCR.**

**
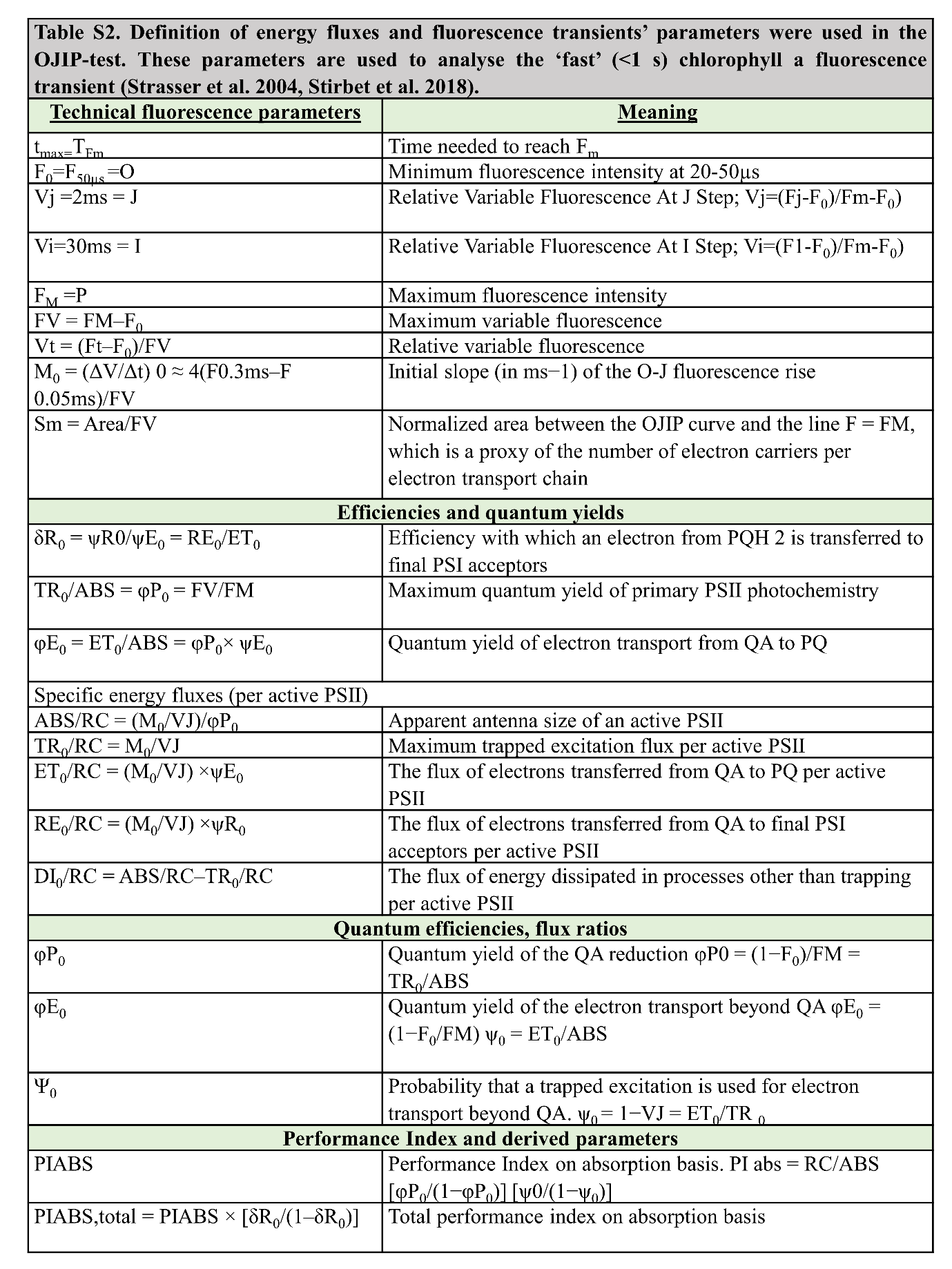
**

**Table S2.** Definition of energy fluxes and fluorescence transients’ parameters were used in the OJIP-test. These parameters are used to analyze the ‘fast’ (<1 s) chlorophyll a fluorescence transient (Strasser et al., 2004).
